# A clinical model to predict the progression of knee osteoarthritis: data from Dryad

**DOI:** 10.1186/s13018-023-04118-4

**Published:** 2023-08-28

**Authors:** Lianwei Shen, Shouwei Yue

**Affiliations:** Rehabitation Center, Qilu Hospital of Shandong University, Jinan, 250000 Shandong China

**Keywords:** Knee osteoarthritis, Clinical prediction models, Dryad

## Abstract

**Background:**

Knee osteoarthritis (KOA) is a multifactorial, slow-progressing, non-inflammatory degenerative disease primarily affecting synovial joints. It is usually induced by advanced age and/or trauma and eventually leads to irreversible destruction of articular cartilage and other tissues of the joint. Current research on KOA progression has limited clinical application significance. In this study, we constructed a prediction model for KOA progression based on multiple clinically relevant factors to provide clinicians with an effective tool to intervene in KOA progression.

**Method:**

This study utilized the data set from the Dryad database which included patients with Kellgren–Lawrence (KL) grades 2 and 3. The KL grades was determined as the dependent variable, while 15 potential predictors were identified as independent variables. Patients were randomized into training set and validation set. The training set underwent LASSO analysis, model creation, visualization, decision curve analysis and internal validation using R language. The validation set is externally validated and F1-score, precision, and recall are computed.

**Result:**

A total of 101 patients with KL2 and 94 patients with KL3 were selected. We randomly split the data set into a training set and a validation set by 8:2. We filtered “BMI”, “TC”, “Hypertension treatment”, and “JBS3 (%)” to build the prediction model for progression of KOA. Nomogram used to visualize the model in R language. Area under ROC curve was 0.896 (95% CI 0.847–0.945), indicating high discrimination. Mean absolute error (MAE) of calibration curve = 0.041, showing high calibration. MAE of internal validation error was 0.043, indicating high model calibration. Decision curve analysis showed high net benefit. External validation of the metabolic syndrome column-line graph prediction model was performed by the validation set. The area under the ROC curve was 0.876 (95% CI 0.767–0.984), indicating that the model had a high degree of discrimination. Meanwhile, the calibration curve Mean absolute error was 0.113, indicating that the model had a high degree of calibration. The F1 score is 0.690, the precision is 0.667, and the recall is 0.714. The above metrics represent a good performance of the model.

**Conclusion:**

We found that KOA progression was associated with four variable predictors and constructed a predictive model for KOA progression based on the predictors. The clinician can intervene based on the nomogram of our prediction model.

**Key information:**

This study is a clinical predictive model of KOA progression. KOA progression prediction model has good credibility and clinical value in the prevention of KOA progression.

## Introduction

Knee osteoarthritis (KOA) is a multifactorial, slow-progressing, non-inflammatory degenerative disease primarily affecting synovial joints. It is usually induced by advanced age and/or trauma and eventually leads to irreversible destruction of articular cartilage and other tissues of the joint [1]. KOA is the musculoskeletal disease with the highest prevalence, and a variety of therapeutic options have been developed for KOA. These include: (i) targeted drugs that inhibit the degradation of articular cartilage and bone matrix [2]; (ii) various anabolic drugs that induce chondrocyte proliferation and cartilage matrix production [3]; (iii) stem cell therapies [4]; (iv) subchondral bone therapies to improve the structure and function of overlying cartilage of the joints [5]; (v) Bisphosphonates to inhibit the activity of osteoclasts and thereby slow down bone turnover [6]; and (vi) supplementation of vitamin D3 to increase the intestinal uptake of calcium and phosphate [6]. Calcium and phosphate uptake by the intestine, thereby improving joint function [7]. Despite the variety of therapeutic approaches, the deterioration of KOA is complex. It involves multiple cytokines, cellular pathways and metabolic pathways [1]. None of the above treatments have been shown to be effective in delaying the progression of KOA. Therefore, studying the factors affecting the progression of KOA may be a new idea for KOA prevention and treatment. It has been found that a variety of predictors such as disease history, medication history, lifestyle, occupation, and demographic characteristics may be associated with the development of KOA [8–11]. Existing articles on KOA progression include bioinformatics-based screening of KOA causative genes and prediction of KOA progression from laboratory-based tests. For example, whole transcriptome gene sequencing results of synovial tissues from KOA patients were downloaded from the GEO database. After the authors took the intersecting genes from weighted correlation network analysis and LASSO regression analysis, the area under the ROC curve was used to verify the gene confidence [12]. Similarly, patient physical examination markers and laboratory markers were used to predict the progression of KOA. After using LASSO regression analysis, the authors found that age, pulse rate, mean hemoglobin concentration, and urea nitrogen could be used for prediction of KOA progression [13]. These new findings have benefited from further developments in molecular biology and testing techniques. However, there is still some difficulty in applying the above findings to the clinic. These findings have great potential in terms of drug therapy for KOA progression, but have limited guidance for clinical wor\k. Therefore, we downloaded the osteoarthritis of the knee data set from the Dryad database, which contains the patient's Kellgren–Lawrence (KL) ratings and many of the factors that allow for clinically effective interventions. Based on logistic regression with LASSO, we used KL2 grade versus KL3 grade as the dependent variable and intervenable clinical factors as the independent variables. We screened the factors that may be associated with KOA progression and constructed a KOA progression prediction model. It provides an effective predictive tool for clinicians to intervene in KOA progression.

## Materials and methods

### Sources of data and topics of study

Dryad is an open data knowledge base that stores medical, biological, and ecological data. It aims to provide an infrastructure for scholarly literature, promote its reuse, and make data from academic papers detectable, freely reusable and quotable [14]. It hosted alternately by the National Center for Ecological Analysis and Synthesis (NCEAS) in California and National Evolutionary Synthesis Center in North Carolina. The data set for this study was downloaded from the article “Assessment of cardiovascular risk factors in patients with osteoarthritis of the knee” [15]. The KL classification is specifically used to assess the severity of KOA. Based on radiographic presentation, patients can be categorized into grades 0–4. This rating is an ordered categorical variable, with grade 0 representing the absence of KOA and grade 4 representing severe KOA [16]. The rating is an ordered categorical variable, with higher scores representing more severe KOA. We included data from patients with ratings KL2 versus KL3 to transform the predictive target of the data set into a dichotomous categorical variable. Inclusion criteria were: KL stage of grade 2 and 3. Exclusion criteria were as follows: samples with missing values.

### Data processing and statistical analysis

**Fig. 1:**
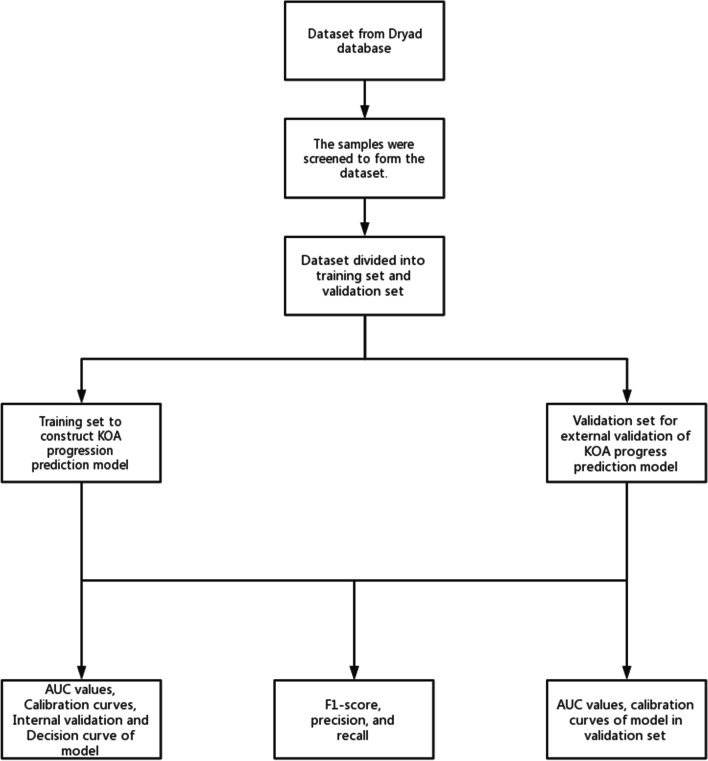
Flow chart of this study

Data processing: 195 patients with KOA were included in the data set based on inclusion criteria and exclusion criteria was performed, including 101 (52%) with KL2 grade and 94 (48%) with KL3 grade. The reasonableness of the data has been verified by the original authors, so no further normalization is required. The data set was randomly split 8:2 into a training set (159 individuals) for model building and a validation set (36 individuals) for validation of the model. Statistical analysis: (i) Model predictors filtering: LASSO allows variables to be selected by setting the coefficient weights on predictors that are irrelevant to the outcome to zero [17]. For the logistic regression with LASSO analysis, we used the “glmnet” package, performing LASSO based predictor filtering on the training set and deriving coefficient scores for each of the predictors. To build the prediction model for KOA progression, the final predictors were filtered with coefficient scores < 0.05. (ii) Clinical prediction model visualisation: The model was visualised using the “nomogram” function in the “rms” package. (iii) Model assessment: The C_index of the KOA progression prediction model was calculated to assess model discrimination. To evaluate the calibration of the KOA progression prediction model, a calibration curve was generated. Evaluate the net benefits of the model using decision curves (DCA). Bootstrap resampling was used to internally validate the KOA progression prediction model. The validation set was used for external validation to verify the discrimination and calibration of the prediction model [18]. Finally, we constructed the confusion matrix in R language and obtained the F1 score, Precision and Recall of the model based on the confusion matrix. A study flow chart is illustrated in Fig. 1.

## Results

### Statistical description of the KOA data set

There were 195 KOA patients in the data set, of which 101 (52%) were KL grade 2 and 94 (48%) were KL grade 3. Comparisons were made between the case and control groups in terms of “B G PRASAD SES”, “BMI”, “TC”, “HDL”, “SBP”, “Hypertension treatment”, “Histor1 of Diabetes Mellitis”, “History of CVD in close to relative < 60 years of age”, “Histor1 of Rheumatoid arthritis”, “Heart Age”, “JBS3 (%)”, and “Life Expectancy” had a different cast, and differences were found to be statistically significant (*P* < 0.05) as indicated in Table 1.

**Table 1:** Statistical description of the data set

Predictors	Group	KL2 level (*n* = 101)	KL3 level (*n* = 94)	*P*
AGE				0.186
		31 (50–55)	23 (50–55)	
		28 (56–60)	25 (56–60)	
		25 (61–65)	22 (61–65)	
		11 (66–70)	14 (66–70)	
		3 (71–75)	8 (71–75)	
		2 (76–80)	2 (76–80)	
		1 (81–85)		
GENDER		Female (57)	Female (56)	0.659
		Male (44)	Male (38)	
B G PRASAD SES				0.006
		Lower middle (23)	Upper (1)	
		Upper lower (43)	Upper middle (11)	
		Lower (35)	Lower middle (24)	
			Upper lower (38)	
			Lower (22)	
BMI		24.8289 ± 2.79	26.39 ± 3.60	0.001
SMOKER		Y (16)	Y (18)	0.545
		N (85)	N (76)	
TC		188.34 ± 32.99	213.66 ± 31.31	< 0.001
HDL		53.74 ± 7.15	51.99 ± 7.44	0.096
SBP		128.57 ± 14.85	139.40 ± 17.52	< 0.001
Hypertension treatment		Y (83)	Y (67)	< 0.001
		N (18)	N (27)	
History of Diabetes Mellitus		Y (4)	Y (38)	< 0.001
		N (97)	N (56)	
History of CVD in a near relative < 60 years of age		Y (5)	Y (16)	< 0.001
		N (96)	N (78)	
History of Rheumatoid arthritis		N (101)	Y (9)	0.001
			N (85)	
Heart age				< 0.001
		26 (50–55)	4 (50–55)	
		13 (56–60)	5 (56–60)	
		26 (61–65)	15 (61–65)	
		20 (66–70)	15 (66–70)	
		6 (71–75)	14 (71–75)	
		5 (76–80)	10 (76–80)	
		3 (81–85)	12 (81–85)	
		2 (86–90)	5 (86–90)	
			4 (91–95)	
JBS3 (%)		11.18 ± 6.56	23.19 ± 14.43	< 0.001
Life expectancy				< 0.001
		5 (71–75)	5 (66–70)	
		26 (76–80)	9 (71–75)	
		51 (81–85)	36 (76–80)	
		18 (86–90)	39 (81–85)	
		1 (91–95)	5 (86–90)	

B G PRASAD SES, Socio economic Status of the patient as per the B G Prasad scale; BMI, Body Mass Index of the patient in kilograms per square meter; TC, Serum Total Cholesterol of the patient in milligram per deciliter; HDL, Serum High density Lipoprotein of the patient in milligram per deciliter; SBP, Systolic Blood Pressure of the patient in millimeters of mercury; Hypertension treatment, Whether the patient is currently on any anti-hypertensive treatment; History of diabetes mellitis, Whether the patient has Diabetes Mellitis; History of CVD in a near relative < 60 years of age:Whether any near relative of age less than 60 of the patient has cardiovascular Disease; History of Rheumatoid arthritis: whether the patient has Rheumatoid Arthritis; Heart Age, Physiological Heart Age of the patient calculated as per JBS3 risk score calculator; JBS3 (%) (10 years risk of developing CVD), Percent risk of developing cardiovascular disease in the next 10 years calculated as per the JBS3 risk score calculator; Life expectancy, Life Expectancy of the patient calculated as per the JBS3 risk score calculator

### Logistic regression with LASSO analysis and model visualization

**Fig. 2:**
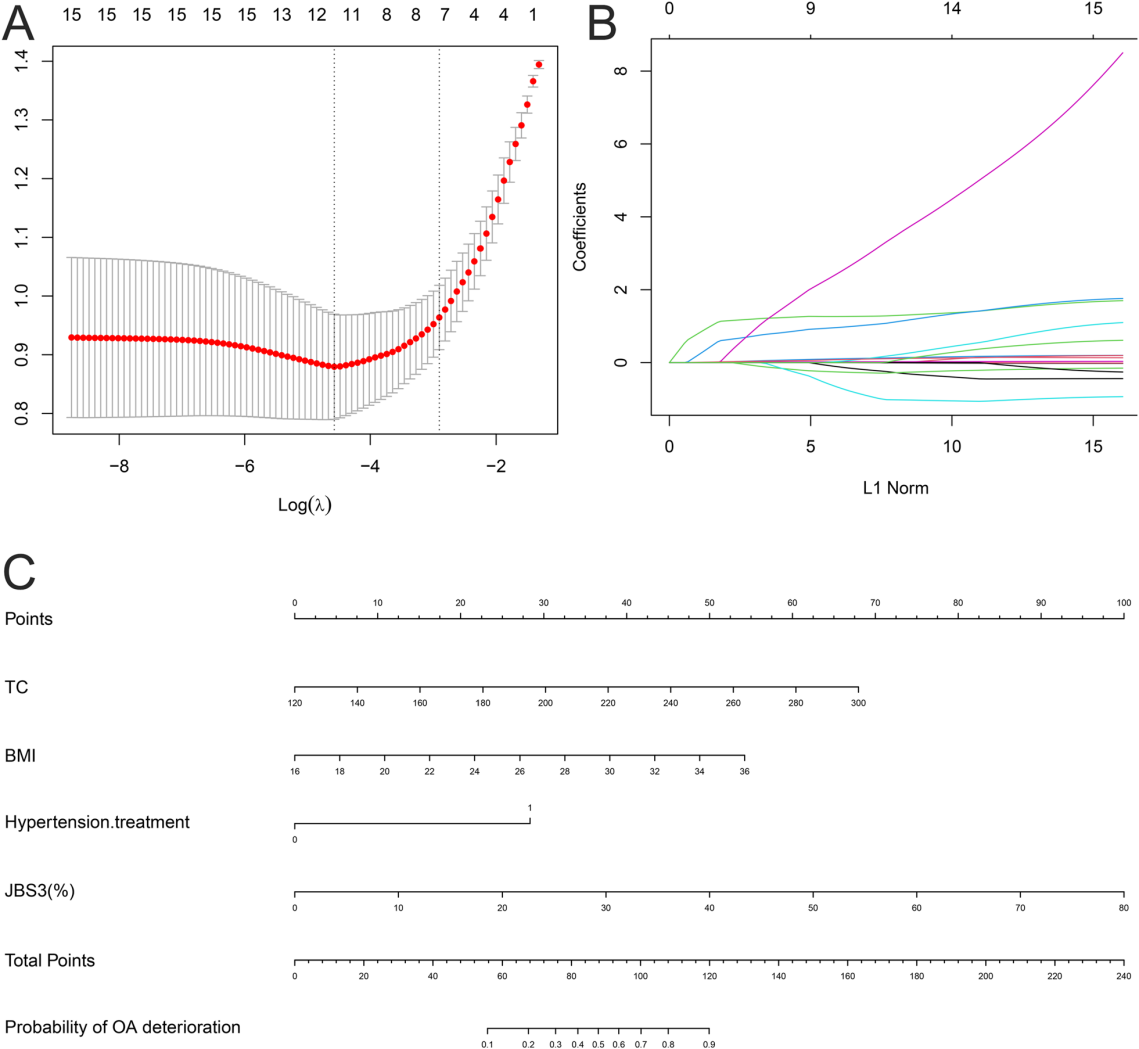
Visualization of logistic regression with LASSO analysis and clinical prediction model. **A** Curve of regression coefficients with Log(*λ*) in LASSO regression The vertical dashed line on the left side of the figure indicates the Log(*λ*) that achieves the minimum value (lambda.1se), and the vertical dashed line on the right side of the figure indicates the Log(*λ*) that is one standard error from the minimum value (lambda.min). **B** Curve of regression coefficients with Log(*λ*), which decreases as the coefficients score continues to go down. **C** Columnar plot of the KOA progression prediction model, with Total Points corresponding to probabilities representing the likelihood of KOA progression

The logistic regression with LASSO analysis is performed on the training set by the “glmnet” package in R (4.2.2). The LASSO path diagram (Fig. 2A) shows two special *λ* values for lambda.min and lambda.1se. The results of this study are presented in Fig. 2B suggests that the predictors decrease with decreasing coefficients. Based on the lambda.1se in Fig. 2A, we we filtered “AGE”, “B G PRASAD SES”, “BMI”, “SMOKER”, “TC”, “HDL”, “Hypertension treatment”, “History of Diabetes Mellitis”, “History of Rheumatoid arthritis”, “History of CVD in a near relative < 60 years of age”, “JBS3 (%) (10 years risk of developing CVD)” for a total of 11 predictors and calculated the coefficients score for each predictor. The selection of “BMI”, “TC”, “Hypertension.treatment”, and “JBS3 (%)” as the final predictor variables to build the prediction model for KOA progression with coefficient score < 0.05 was significant. The “nomogram” function of the “rms” package was used to create a nomogram based on the KOA progression prediction model (Fig. 2C). The application of the nomogram is as follows: according to the nomogram, the Points corresponding to each predictor of an individual are derived, and the Total Points of all predictors are derived. After the Total Points of all predictors are obtained, the prediction probability corresponding to the Total Points is the probability of KOA patient progression.

### Efficacy of the KOA progression model

**Fig. 3:**
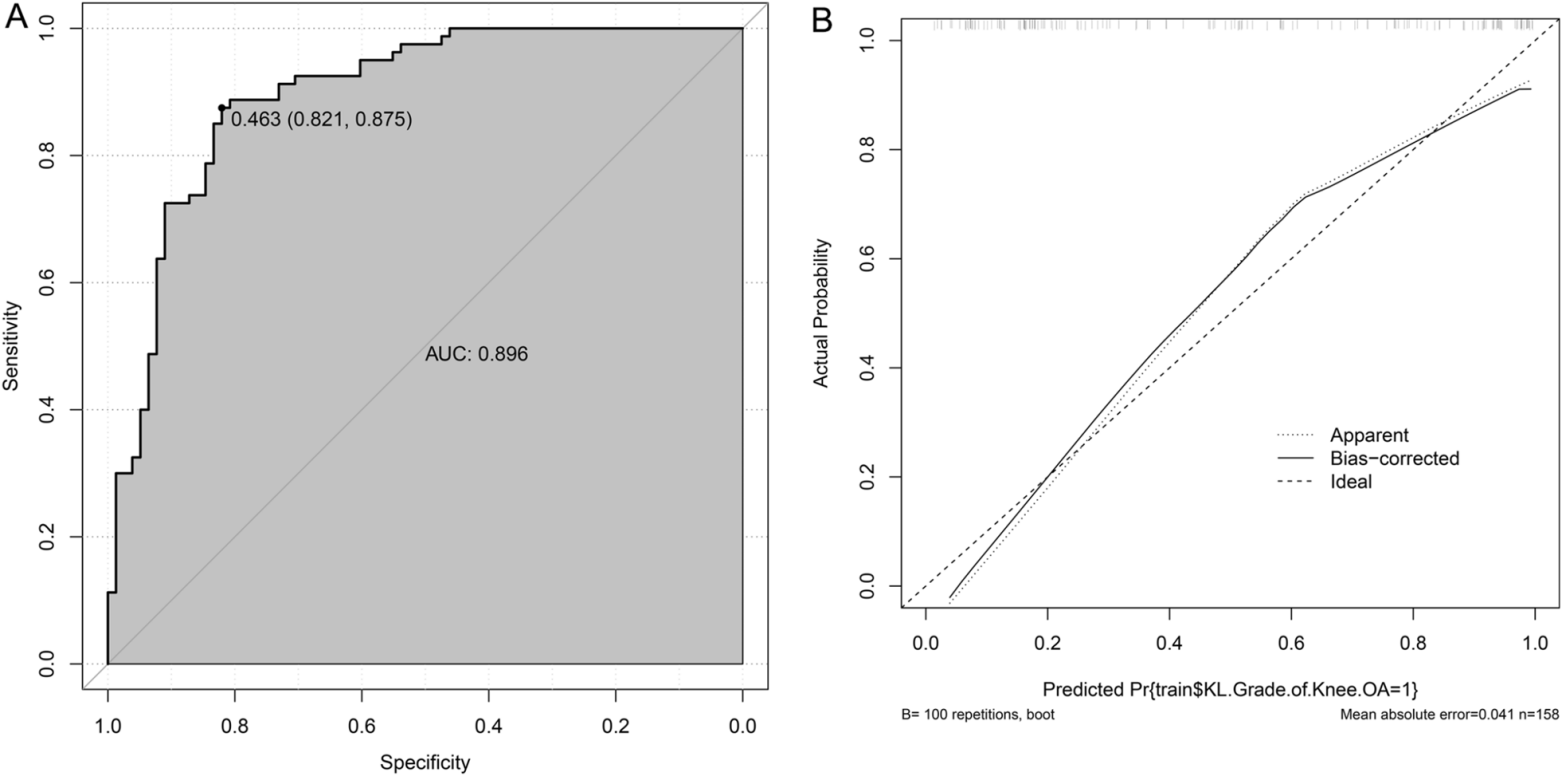
Efficacy of KOA progression model. **A** ROC curve of the model, **B** Calibration curve of the model

The ROC curve and calibration curve for the KOA progression prediction model were graphed in the R language. The area under the model's ROC curve (Fig. 3A) is 0.896 (95% CI 0.87–0.945), which corresponds to the area under the curve, indicating that there is a high degree of discrimination in the model. The value of the optimal cutoff is 0.463 (95% CI 0.821–0.875). The MAE of the standard curve is 0.041, which indicates that the model has a large degree of calibration (Fig. 3B).

### Internal validation of the KOA progression model

**Fig. 4:**
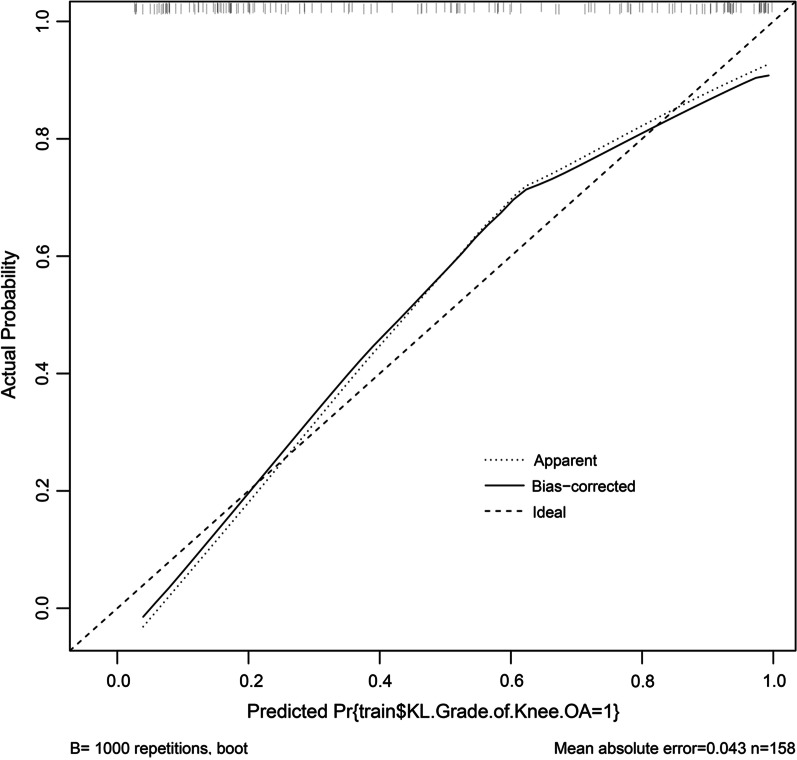
Internal validation of the KOA progression model

The Bootstrap method was used for the internal validation of the training set, and the number of resampling number was set to 1000. The calibration curve MAE plotted was 0.043, which indicates that the model has a high degree of calibration (Fig. 4).

### External validation of the KOA progression model

**Fig. 5:**
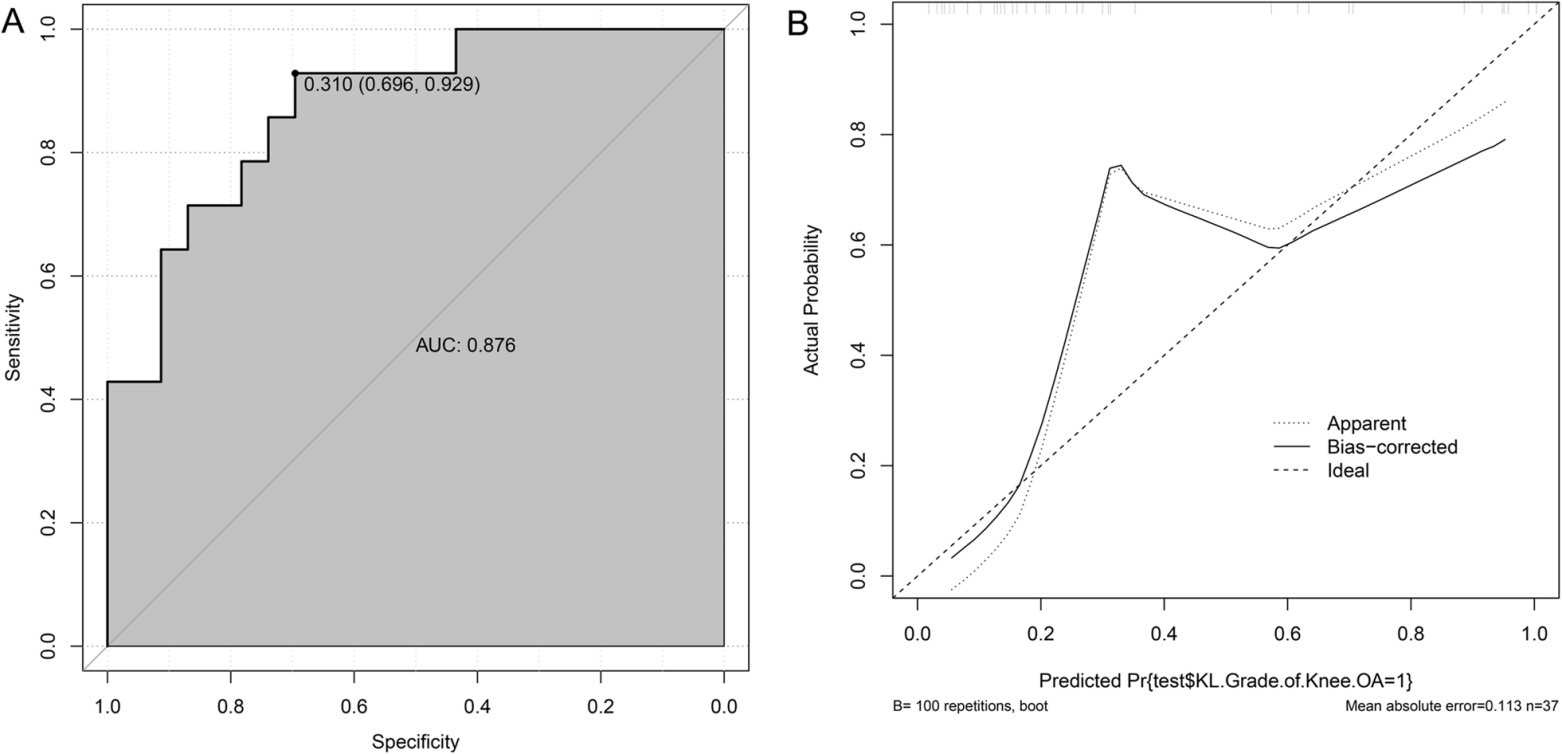
External validation of KOA progression model. **A** ROC curve for external validation, **B** Calibration curve for external validation

The external validation of the model in R language based on the final predictors in the validation set yielded an area under the ROC curve (Fig. 5A) of 0.876 (95% CI 0.767–0.984). It indicating that the model has a high degree of discrimination, and the optimal cutoff value is 0.310 (95% CI 0.696–0.929). The calibration curve MAE is 0.113, indicating that the model has a high calibration degree (Fig. 5B).

### DCA for KOA progression model

**Fig. 6:**
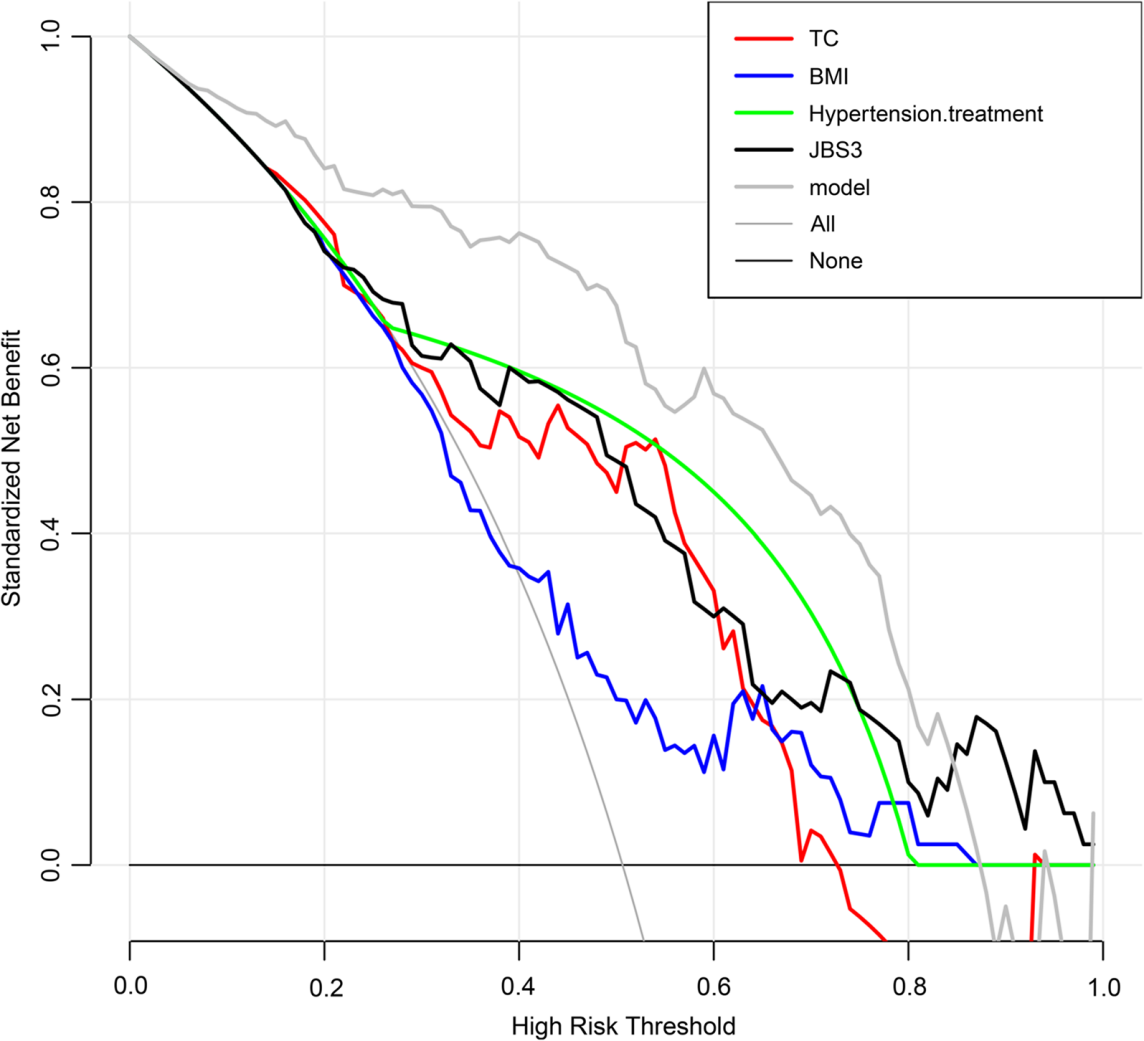
DCA of KOA progression model

The DCA for KOA progression, the horizontal coordinate represents the threshold probability, which can be interpreted as the number of samples greater than this value/total number of samples after clustering by a KL classifier. The vertical coordinate is the net gain, which is the relative gain derived by subtracting the proportion of true positive results from the proportion of false positive results weighted by the threshold probability ratio. None and ALL are the two reference lines, and the closer the model curve made by different predictors is to the two reference lines, the less it has application value. The higher the vertical coordinate in the same horizontal coordinate case means the better the model. The model made up of 4 predictors in the values of the vertical coordinates in a large (horizontal coordinate) threshold interval are higher than those of the model constructed by a single predictor model. It indicating that the prediction model constructed in this study has a high degree of confidence (Fig. 6).

### F1-score, precision, and recall

Precision is the proportion of true positive cases that are predicted to be positive by the model. Recall is the proportion of true positive cases that are predicted to be positive by the model. The score is the reconciled mean of precision and recall, which is used to evaluate the performance of the model together. All three take values between 0 and 1, with closer to 1 indicating better model performance. In this study, Precision is 0.667, recall is 0.714, and F1_score is 0.690. This indicates that the model performs well in determining true positive instances, finding true positive instances, and model performance.

## Discussion

Based on a systematic evaluation of osteoarthritis, it appears that KL grade is the most commonly used indicator of KOA progression. Combination of joint structure and clinical risk factors may be the best combination for the prediction of KOA progression [19]. Therefore, in this study, a data set of KOA was downloaded from the Dryad database. And a clinical prediction model of KOA progression was constructed by incorporating multiple risk factors that may be associated with KOA progression, using a patient KL grade of 2 or 3 as an outcome indicator. The AUC of the predictive model is an index for measuring the classification accuracy of the model [18]. A higher value indicates better model accuracy. The AUC of the KOA model was 0.896 (95% CI 0.847–0.945). It indicates that the prediction model has a high accuracy and is able to distinguish between KL2 and KL3. MAE is a measure used to measure model prediction errors. MAE represents the mean absolute error of the residual in the calibration curve, that is, the mean of the difference between predicted and actual values. As the MAE becomes smaller, the model's predictive power improves [18]. MAE of the calibration curve was 0.041, indicating that the model has a high degree of calibration. The MAE of the internal validation is 0.043, which indicates that the model also has high consistency. The external validation of the KOA prediction model with an area under the ROC curve of 0.876 (95% CI 0.767–0.984) by the validation set. It demonstrated the strong diagnostic ability of the KOA model as well as the ability to accurately discriminate between the levels of KL2 and KL3 in a completely novel data set. The calibration curve of the Mean Absolute error is 0.113, indicating that the KOA model has a higher calibration in the new data set. F1-score, Precision, and Recall also indicate that the model has good performance. The KOA model made by “BMI”, “TC”, “Hypertension.treatment”, and “JBS3 (%)” has high reliability. It can accurately predict the probability of KOA progression from grade 2 to grade 3 based on the above predictors, and provide a reference for the prevention of KOA progression. As can be seen from the nomogram, BMI is positively correlated with KOA progression, and the higher the BMI, the greater the likelihood of KOA progression. One of the major risk factors for KOA is obesity [20, 21]. Research has shown that knee osteoarthritis is associated with excessive loading of the joint [20], which is manifested as an inflammatory reaction, pain, and swelling of the knee joint [21]. Studies have shown that obesity increases knee joint loading. This results in obese KOA patients performing worse than non-obese KOA patients in terms of functional mobility, gait speed, pain and activities of daily living [22]. Obesity is also considered a state of chronic low grade inflammation, which may promote the generation of oxidized serum low density lipoprotein (LDL) [23, 24]. Oxidized LDL is believed to play a significant role in KOA production, which is associated with free movement of oxidized LDL in and out of the knee joint [25]. Elevated levels of oxidized LDL activate macrophages, fibroblasts, and endothelial cells in the synovial tissue of the knee, leading to local inflammation, apoptosis of chondrocytes, and ectopic ossification in the knee joint [26]. This is in contrast to the prediction model for KOA progression, which suggests that high blood fat disease may also be a risk factor for progression of KOA. And how high blood fat disease promotes the development of KOA requires further study. The nomogram of the KOA prediction model shows that receiving treatment for hypertension is a risk factor for progression of KOA. This does not suggest that treatment of hypertension is harmful for KOA patients, but rather that hypertension is a risk factor for KOA progression. A large number of cross-sectional and longitudinal cohort studies have reported an association between KOA and hypertension [27–29]. Patients with KOA have pathological alterations in the extracellular matrix that result in reduced vascular elasticity and thereby promote hypertension [29, 30]. Also, patients with KOA often exhibit a chronic inflammatory state, which may also play a role in the generation and progression of hypertension [31]. The Joint British Society QRisk3 calculator is a comprehensive risk score calculator. It calculates the 10 years risk of developing cardiovascular disease, physiological heart age, and life expectancy as a function of age, body mass index, systolic blood pressure, diabetes, total cholesterol, high density lipoprotein and smoking status [32]. In a cross-sectional study of KOA and cardiovascular risk factors, KOA was strongly associated with CVD, with variable 10 years CVD risk positively correlated with KOA severity [31]. The KOA risk factors noted above do not independently affect the progression of KOA. Patients with Obese KOA who have high joint loads are more likely to present with joint pain, and joint pain decreases physical activity in patients with KOA, making them more susceptible to CVD [33]. Obesity is a chronic inflammatory state that is often accompanied by multiple pathophysiological process such as sympathetic nervous system arousal, increased endothelial oxidative stress, increased arterial stiffness, and cardiac remodeling. They together result in an increase in blood pressure in patients with KOA [34]. Limitations of our study include: (i) it is a single center study, and a more representative multi-centre sample must be included; (ii) predictors do not implicate the genetic level, and model efficiency can be further improved if patient genomic sequencing data can be added as predictor variables.

## Conclusion

Briefly, we constructed a prediction model of KOA progression with high confidence. This model allows clinicians to intervene in weight control, lipid lowering, blood pressure control, and monitoring of CVD in patients with KOA in order to slow the progression of KOA.

## Data Availability

The data that support the findings of this study are available in Dryad at 10.5061/dryad.79cnp5htv, reference number: 10.5061/dryad.79cnp5htv. These data were derived from the following resources available in the public domain: Goel, Sagar (2021), Assessment of cardiovascular risk factors in patients with Knee Osteoarthritis, Dryad, Dataset, 10.5061/dryad.79cnp5htv.

## Abbreviations

KOA: Knee osteoarthritis
DCA: Decision curves
KL: Kellgren–Lawrence
MAE: Mean absolute error
NCEAS: National Center for Ecological Analysis and Synthesis
